# Desmoplastic Cutaneous Squamous Cell Carcinoma Is an Aggressive and Potentially Fatal Skin Cancer: A Systematic Review

**DOI:** 10.1111/cup.70079

**Published:** 2026-03-15

**Authors:** Andrew Dettrick, Haris Mohammed, Thomas Hardman, Nicole Buddle, Ryan Livingston, Duncan Lambie, Rebecca Donkin

**Affiliations:** ^1^ Pathology Queensland Sunshine Coast Australia; ^2^ School of Health University of the Sunshine Coast Sippy Downs Australia; ^3^ Sullivan Nicolaides Pathology Sunshine Coast Australia; ^4^ School of Medicine and Dentistry Griffith University Sunshine Coast Australia; ^5^ Townsville University Hospital Townsville Australia; ^6^ Sunshine Coast University Hospital Birtinya Australia; ^7^ Sullivan Nicolaides Pathology Brisbane Australia; ^8^ Frazer Institute University of Queensland Brisbane Australia; ^9^ Pathology Queensland, Princess Alexandra Hospital Brisbane Australia

**Keywords:** carcinoma, histopathology, skin neoplasms, squamous cell

## Abstract

The incidence and mortality of cutaneous squamous cell carcinoma (cSCC) are increasing. Desmoplastic cSCC (dcSCC) is an uncommon yet highly aggressive variant which has received relatively little attention in the literature. No systematic reviews of dcSCC are currently available. This paper aims to summarize the current understanding of the behavior of dcSCC by conducting a systematic review of published series with particular focus on outcomes. A comprehensive search was conducted for original series in English. Six studies reporting a total of 286 dcSCC were included in the final analysis. Most series were single site and retrospective. Pooled estimates demonstrated local recurrence rate 30% (95% CI: 24%–36%, *p* = 0.765), nodal metastasis 17% (95% CI: 12%–22%, *p* = 0.158), distant metastasis 5% (95% CI: 2%–9%, *p* = 0.574) and disease‐specific death 12% (95% CI: 3%–36%, *p* = 0.047), outcomes which are considerably worse than non‐dcSCC. The findings confirm the aggressive nature of dcSCC and suggest early aggressive treatment is warranted. However, given the limitations of existing studies, there is a need for large, prospective, multicentre studies to better define prognostic factors and optimize management strategies.

## 1 Introduction

### 1.1 Rationale

The incidence of cutaneous squamous cell carcinoma (cSCC) is increasing worldwide, resulting in an estimated 64 000 deaths in 2020 [1]. As the second most common human malignancy, cSCC is extremely common; however, the majority of cases are identified early and easily cured with local therapies [2]. However, a minority subset of cases show aggressive behavior, which can result in local recurrence, metastasis, and death [3]. The prognosis of cSCC is strongly influenced by stage, with survival falling significantly once metastasis has occurred [4]. Therefore, identifying and treating high‐risk tumors early is critical to improving outcomes [5].

Considerable efforts have been made to identify features which predict poor outcomes for cSCC. High‐risk features include both patient‐related factors such as immunosuppression and neurological symptoms and tumor‐related factors such as large tumor size, histological poor differentiation, deep invasion, perineural invasion (PNI), satellitosis/in transit metastasis (SITM), lymphatic or vascular invasion (LVI), and the desmoplastic cutaneous squamous cell carcinoma (dcSCC) subtype [5, 6]. The dcSCC subtype itself is strongly predictive of a poor outcome, with the NCCN Clinical Practice Guideline classifying it as “Very High Risk” and recommending more aggressive local treatment such as adjuvant radiotherapy and more frequent follow‐up [6].

**FIGURE 1:**
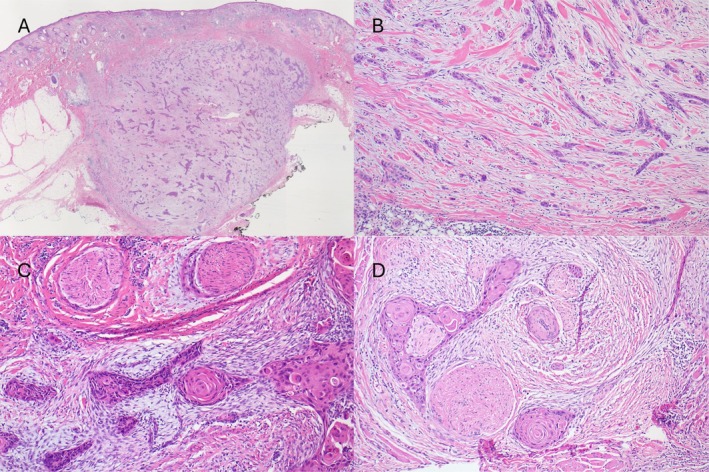
Desmoplastic cutaneous squamous cell carcinoma characteristic features. (A) Typical low power appearance of dcSCC (H&E orig mag ×10); (B) Fine branches of tumor cells at the periphery; (C) Squamous differentiation is usually obvious and most tumors are not poorly differentiated using classic Broders grading, note PNI also present; (D) DcSCC is strongly associated with PNI (C, D, H&E orig mag ×100).

**FIGURE 2:**
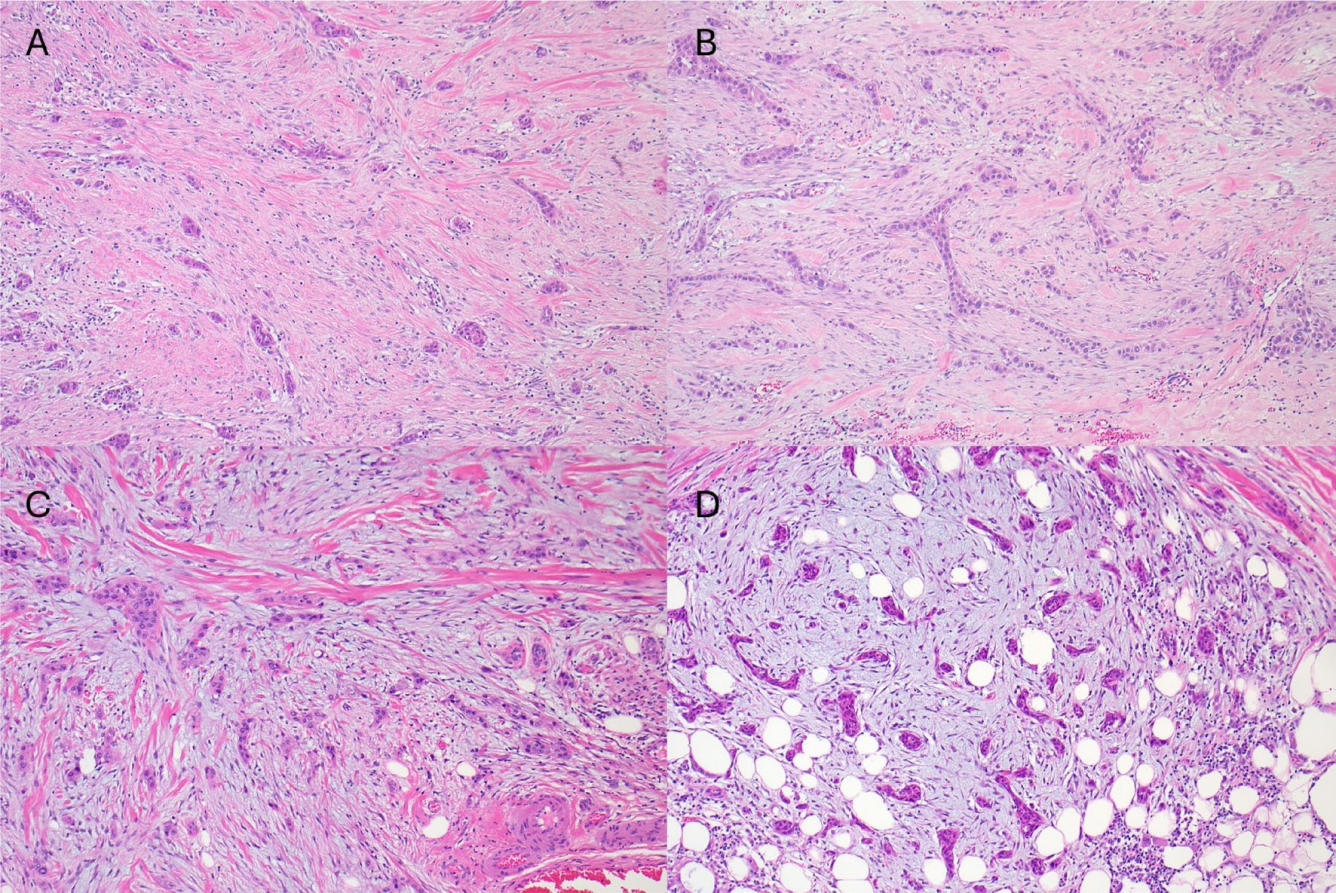
The range of appearances of the eponymous desmoplastic stroma: (A) Dense sclerotic stroma; (B) Fibrous stroma at a slightly earlier stage with less mature collagen; (C) Fibromyxoid stroma; (D) Immature myxoid stroma; (all H&E orig mag ×100).

DcSCC can be defined as a primary cSCC characterized by fine branches of tumor cells at the periphery and a surrounding desmoplastic stromal reaction in at least a third of the tumor [7, 8]. Some authors have proposed 50% desmoplasia as the cut‐off [9, 10]. The concept of desmoplastic tumor stroma is familiar to pathologists and, in the context of dcSCC, may be defined as a tumor‐related stromal reaction demonstrating fibroblast activation and dense sclerotic stroma, early fibrous stroma or myxoid stroma—see Figures 1 and 2.

DcSCC was first described in a case report in 1989 (published in German) [7]. The first series, published in 1997, demonstrated dcSCC is 10 times more likely to recur locally and 6 times more likely to metastasise compared to conventional cSCC [8]. The World Health Organization Classification of Skin Tumors subsequently included dcSCC as a specific variant of conventional cSCC, and it was included in skin cancer clinical practice guidelines [6, 11]. Despite this recognition, dcSCC remains relatively underappreciated in clinical practice as it is often grouped with conventional cSCC and there is no distinctive management pathway at the time of writing. DcSCC has received relatively little attention in the literature; a preliminary search of Pubmed, Web of Science and PROSPERO was performed on 21 October 2025 and no systematic reviews were identified.

## 2 Objective

This study aims to highlight this potentially fatal cancer by systematically analyzing the outcomes reported in published series of dcSCC, focusing on local recurrence (LR), nodal metastasis (NM), distant metastasis (DM), and disease‐specific death (DSD). Given its high‐risk nature, this study seeks to provide a clearer understanding of dcSCC behavior and prognosis and emphasize the need for early identification and distinct management approaches compared to non‐dcSCC.

## 3 Method

The study followed the PRISMA 2020 guidelines [12].

### 3.1 Eligibility Criteria

Studies were included if they focused primarily on dcSCC with follow‐up data on at least one of the following prognostic indicators: LR, NM, DM, or DSD. Only original series in English were included. Case reports, conference abstracts, and studies with overlapping patient populations were excluded.

### 3.2 Information Sources and Search Strategy

A comprehensive search of PubMed and Web of Science was performed on 20 October 2024 for the following terms: (skin OR cutaneous) AND (“squamous cell carcinoma”) AND (desmoplastic OR sclerosing).

### 3.3 Selection Process

Two independent reviewers screened title and abstract. Full‐text screening was then performed to ensure studies met the eligibility criteria. Disagreements were resolved through discussion.

### 3.4 Data Collection Process

Data extraction was carried out using a pre‐designed data extraction sheet. The following variables were collected:

- Study characteristics: Author, publication date, study design, country.
- Patient demographics: Number of cases, male‐to‐female ratio, median age, age range, immunosuppression status
- Tumor characteristics: Location (head and neck vs. other), tumor size, thickness/depth, anatomical level of invasion, LVI, SITM, PNI, histologic grading (Broders' grading), worst pattern of invasion (WPOI).
- Outcomes: Follow‐up period, LR, NM, DM, DSD, overall survival.

Data extraction was performed independently by two reviewers, and any discrepancies were resolved by discussion. Extracted data were compiled into Microsoft Excel, and statistical analysis was conducted using R.

### 3.5 Data Items

The primary outcomes of interest were LR, NM, DM, DSD. Secondary measures such as patient demographics and tumor characteristics mentioned previously were also collected.

### 3.6 Risk of Bias Assessment

The Critical Appraisal Skills Programme (CASP) qualitative checklist was used to appraise quality depending on the study design—see Table S1 [13].

### 3.7 Effect Measures

Proportions were calculated and pooled proportions were generated for each primary outcome measure.

### 3.8 Synthesis Methods

A meta‐analysis of pooled proportions was performed using R to estimate the rate of LR, NM, DM, DSD. The random‐effects model was applied to account for inter‐study heterogeneity. Forest plots were generated to visualize pooled estimates. To assess heterogeneity, *I* ^2^ values were calculated, and funnel plots were used to evaluate publication bias.

## 4 Results

**FIGURE 3:**
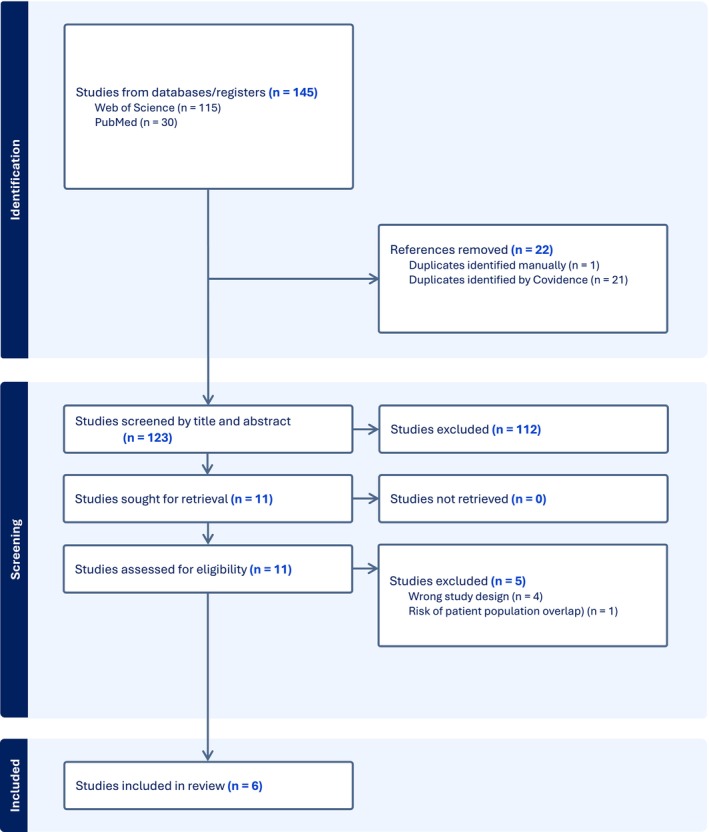
Literature search flow chart.

The initial search returned 145 results and after removal of duplicates 123 studies remained. After title and abstract screening and exclusion of reports that did not meet all inclusion criteria,18 publications underwent full‐text review. Six studies were included in final analysis—see flow chart in Figure 3 [8, 9, 10, 14, 15, 16]. Of the 18 full‐text articles reviewed, 9 were authored by the same research group, leading to the exclusion of several papers due to suspected overlap in patient populations [17, 18, 19, 20, 21, 22, 23].

The included studies described 286 dcSCC in 285 patients (range 6–145)—see Table 1. Two were prospective cohort studies and 4 were retrospective. DcSCC occurred predominantly in men (67%) of advanced age (range of median ages 66–80 years) although it affected a wide age range (31–99 years). Most tumors were located on the head and neck (92%, 256/279) (Table 2).

**TABLE 1:** Study characteristics—Published series of dcSCC.

Source	Study years	Locations	Study type	No. of dcSCC	Sex (M:F)	Age range (years)	Site on head and neck	Follow‐up period (median, months)	Other comments
Breuninger 1997 [9]	1982–1992	Germany, Single site	Prospective cohort	44	29:15	NA	40	64	
Velazquez 2010 [8]	Not stated	United States, Single site	Retrospective cohort	6	5:1	54–86	6	19	Only included unusual cases which mimicked scar
Salmon 2011 [13]	1988–2008	New Zealand and United States, 2 sites	Retrospective cohort	73	46:26	45–91	66	36	Results differ from the rest of the literature.
Ronen 2018 [15]	2013–2017	United States, single site	Retrospective cohort	11	8:3	58–84	10	NA	Includes 5 recurrences in same patient; Data extracted for primaries only
Botha 2019 [9]	2006–2013	Australasia, 3 sites	Retrospective cohort	7	4:3	49–84	NA	48	Periorbital cases only
Haug 2020 [16]	2005–2015	Germany, single site	Prospective cohort	145	98:47	31–99	134	36	
Total				286	190:95 (67% Male)	31–99	256/279 (92%)		

Abbreviation: NA, not available.

**TABLE 2:** Association of dcSCC with high‐risk features.

Source	No. of dcSCC	Immuno‐suppression	Size range (mm)	Level 5 or deeper	LVI	SITM	PNI	Poorly differentiated
Breuninger 1997 [17, 18, 19]	44	NA	NA	NA	NA	NA	NA	12/44
Velazquez 2010 [19, 24, 25]	6	3/6	11–50	5/6	1/6	2/6	1/6	NA
Salmon 2011 [26]	73	NA	5–54	16/67	0/73	NA	53/73	4/69
Ronen 2018 [15]	11	3/11	7–35	5/11	0/11	NA	5/11	NA
Botha 2019 [21]	7	1/7	4–31	NA	NA	NA	NA	NA
Haug 2020 [16]	145	39/145	NA	74/145	NA	5/145	21/145	15/141
Total	286	46/169 (27%)	4–54	100/229 (44%)	1/90 (1.2%)	7/151 (4.6%)	80/235 (34%)	31/254 (12%)

Abbreviations: LVI, lymphovascular invasion; NA, not available; Level 5 or deeper refers to Clark level 5 or deeper; PNI, perineural invasion; SITM, satellitosis/in‐transit metastasis.

Among 169 cases with available data, 27% were immunosuppressed (46/169)—see Table 3. Tumors ranged in size from 4 to 54 mm and 44% are Clark level 5 or deeper (100/229). LVI status was only recorded in 3 series and was present in only 1 case (of 90). PNI appears to be strongly related to dcSCC with this feature present in 34% of cases (80/145). However, this result is strongly influenced by one series which recorded PNI in 53/73 cases. In another series of both dcSCC and non‐dcSCC, PNI was only identified in dcSCC [16]. Most dcSCC were not poorly‐differentiated with only 12% classified as such (31/254).

**TABLE 3:** Outcomes of dcSCC.

Source	No. of dcSCC	LR	NM	DM	DSD	Other outcome measures or comments
Breuninger 1997	44	12/44	10/44	0/44	NA	Compared to non‐dcSCC lesions were thicker and deeper; NM ×6; LR ×10; NM more common in 2‐5 mm group
Velazquez 2010	6	2/6	1/6	1/6	2/6	
Salmon 2011	73	22/58	0/73	NA	0/72	
Ronen 2018	11	2/7	1/7	0/7	0/7	
Botha 2019	7	2/7	0/7	0/7	0/7*	*One patient treated with palliative radiotherapy but outcome not stated
Haug 2020	145	39/145	24/145	6/145	20/76	Progression free survival 50% (vs 89% for non‐dcSCC) and 17% if also PNI. PNI seen exclusively in dcSCC. 21/145 PNI. Diameter and thickness greater. 54/145 progressed (35%)

Abbreviations: DM, distant metastasis; DSD, disease specific death; LR, local recurrence; NA, not available; NM, nodal metastasis; PNI, perineural invasion.

Parenthetically, there is very little written about the clinical appearance of dcSCC and the features do not appear to be distinctive. Reports describe a variety of appearances ranging from scaly erythematous plaques to raised firm nodules [9, 15]. Lesions may be large and ulcerated or may be mistaken for sclerosing basal cell carcinoma or desmoplastic melanoma [10, 24]. In the authors' personal experience, clinical features of dcSCC are not distinct from those of conventional cSCC.

**FIGURE 4:**
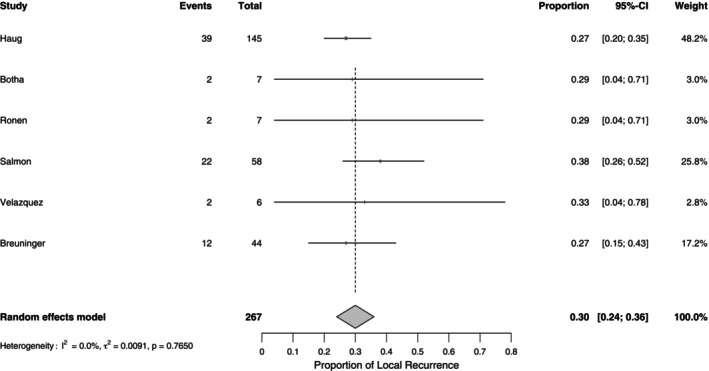
Forest plot for local recurrence in the included studies.

**FIGURE 5:**
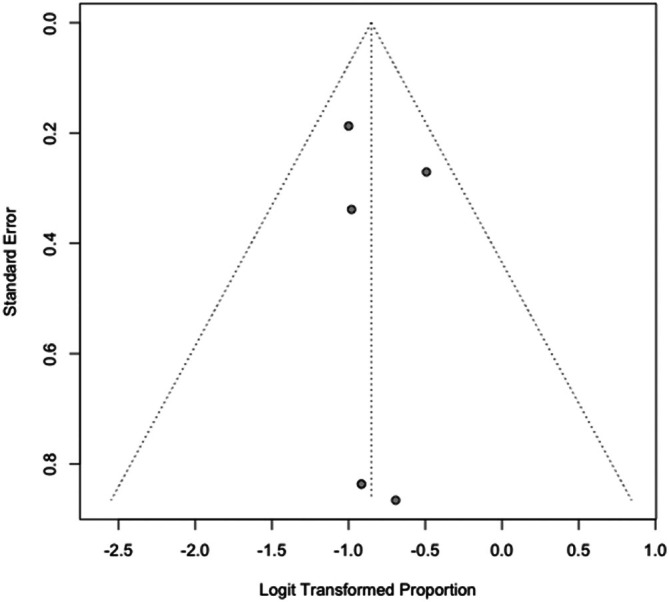
Funnel plot for studies included in pooled proportions in local recurrence.

**FIGURE 6:**
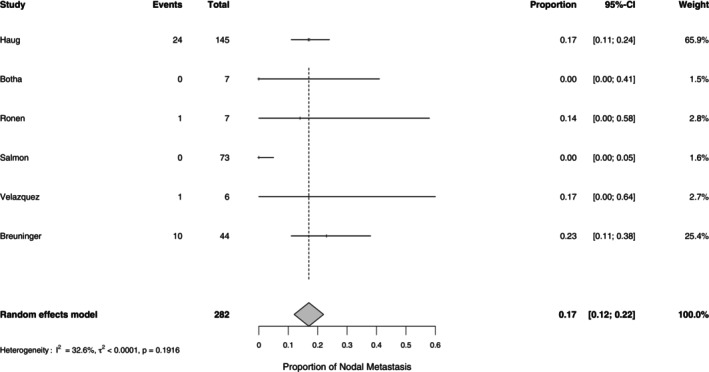
Forest plot for Nodal Metastasis in the included studies.

**FIGURE 7:**
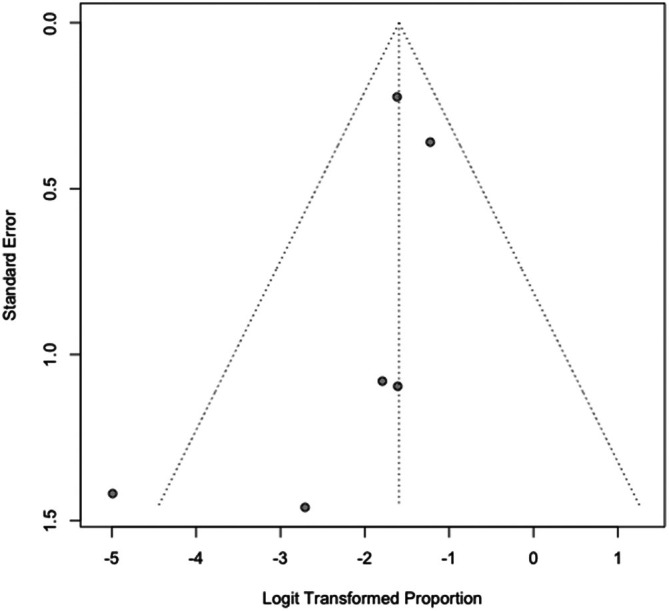
Funnel plot for studies included in pooled proportions in nodal metastasis.

The pooled proportion of local recurrence in dcSCC was 30% (95% CI: 24%–36%; range 27%–38%) with low heterogeneity (*I* ^2^ = 0.0%, *p* = 0.765), indicating a consistent recurrence rate across studies. See Table 3 and Figures 4 and 5. Nodal metastasis was observed in 17% (95% CI: 12%–22%; range 0%–23%) of cases, with moderate heterogeneity (*I* ^2^ = 32.6%, *p* = 0.158). See Figures 6 and 7.

Distant metastasis only occurred in 7/209 tumors for a pooled proportion of 5% (95% CI: 2%–9%) and only occurred in 2 of 5 series, although there was statistically minimal heterogeneity (*I* ^2^ = 0.0%, *p* = 0.574). See Figures S1 and S2.

Disease specific death pooled rate was 12% (95% CI: 3%–36%) however the rate varied considerably across studies with moderate heterogeneity (*I* ^2^ = 59.9%, *p* = 0.047). See Figures S3 and S4. Haug et al. reported DSD in 26% (20/76) while 3 series recorded no deaths [9, 10, 15, 16]. Notable among the later was Salmon et al. who recorded no deaths in the second largest series of 73 cases [10]. The significance of these findings is therefore interpreted with caution.

## 5 Discussion

This review supports the notion of the aggressive locoregional behavior of dcSCC. LR occurred in 30% and NM in 17% in comparison to non‐dcSCC which shows LR about 3%–8% and NM about 4%–5% [19, 25, 26]. The findings underscore the need for obtaining local control followed by vigilant post‐treatment surveillance of the tumor bed and support the need for radiological staging of the nodal bed. Parenthetically, treatment guidelines also suggest consideration of sentinel lymph node biopsy; however, detailed discussion of that topic is beyond the scope of this paper.

Distant metastases were uncommon, being found in only 2 series and only 7/209 tumors overall, hence it appears dcSCC primarily exhibits locoregional spread rather than distant dissemination. Rate of DSD was estimated as 12% compared to 1.5%–3% in non‐dcSCC [19, 27, 28]. The significance of both these findings is somewhat circumspect. Whilst the calculated risk of death from dcSCC appears to be about 4–8× higher than non‐dcSCC, the numbers are too small to draw firm conclusions. Larger, prospective, multicentre studies are required.

It is impossible to accurately count the number of cases of dcSCC that have been published because of overlap between study populations in several of the largest series but the total number of published cases in series that do not overlap is only about 500 [7, 8, 10, 14, 15, 16, 19, 24, 29, 30, 31, 32, 33, 34]. The average incidence of dcSCC as a proportion of all cSCC in the 10 series that include incidence data is 8.3%, applying a weighted mean due to high heterogeneity, with a range of 3.9%–40%, although some of these had overlapping populations [8, 16, 17, 19, 21, 23, 29, 30, 31, 33]. This relatively high proportion, combined with the very high incidence of cSCC, highlights the dearth of publications.

Desmoplasia has been studied in a range of other tumors and appears to be the result of peritumoral stromal remodeling although the underlying mechanism is not well understood [35]. One study of dcSCC proposed that desmoplastic stroma may be an expression of epithelial‐mesenchymal transition [32].

Why dcSCC behaves more aggressively is also uncertain, although several theories have been offered. Some authors have reported that dcSCC is associated with other well‐known high‐risk features including immunosuppression, increased thickness, increased size, SITM and PNI [16, 36, 37, 38]. We found similar associations in this review—see Table 3. One study found a staggering 71% progression rate for those dcSCC which also featured PNI [16]. Regardless of these associations with other high‐risk features, desmoplasia was independently associated with LR and NM in the largest and most comprehensive series [16]. It may be that the highly infiltrative pattern which is characteristic of dcSCC means it is more likely for tumor to be left behind during excision or to be missed during histological examination [39]. It has been suggested that fibroblast activation may be a characteristic of relatively advanced local tumor stage [8, 15]. Another author posited that myxoid stroma may inhibit the host immune response [32].

Interestingly, most dcSCC are not poorly differentiated, with only 12% recorded as such. In this review, the histological grades of the tumors as proffered by the original authors were accepted, but only 3 of 6 articles included unequivocal grading information. Velasquez et al. described cases of spindle cell dcSCC with areas “largely devoid of significant cytological atypia” [14]. This presented a dilemma, as spindle cell SCC is poorly differentiated by definition according to the WHO classification; however, the classification also states that spindle cell SCC “is composed of closely packed fascicles of pleomorphic spindle cells, with frequent mitotic activity.” Due to this ambiguity, the 6 cases described by Velasquez et al. were excluded from our analysis of grading.

Interobserver agreement for the grading of conventional cSCC is known to be weak and there are no published articles regarding interobserver agreement for the grading of dcSCC specifically [39]. Indeed, agreement between pathologists when making the diagnosis of dcSCC appears to be disappointing, although there is only a single abstract published [40]. Hence, it appears the diagnosis and grading of dcSCC are likely to suffer from poor interobserver agreement and more robust and reproducible criteria are needed. It is hoped that the current paper will encourage more studies in this area.

In their series of 73 primary dcSCC, Salmon et al. found no metastases or deaths at 36 months follow‐up [10]. This was despite identifying PNI in 53 cases (73%) and an LR rate of 80% in those cases treated with standard excision, findings which are at odds with the rest of the literature. Their cohort was community‐based and perhaps this represents a selection bias or at least is different to the population in some other series which were drawn from a university hospital [10, 16].

The terminology for dcSCC has not been consistent and series are not necessarily directly comparable [41]. For example, the first report to coin the term “desmoplastic cutaneous squamous cell carcinoma” was Haneke in 1989; however, in 1986 Frierson and Cooper published a series of 187 SCC of the lower lip and defined a subgroup with a dispersed pattern of invasion; 13% of their cases had the dispersed pattern and 77% of those patients developed metastases [7, 42]. Those cases may be equivalent to dcSCC, although it is acknowledged that it is not clear if these represent cases of SCC from the mucosal lip or cutaneous lip.

It is tempting to draw parallels between desmoplastic melanoma and dcSCC; however, there is little to recommend this approach. Both show a tendency to PNI, and both are characterized by abundant collagenous stroma. However, desmoplastic melanoma features highly characteristic, relatively bland, spindle‐shaped tumor cells that are different from other melanomas, while dcSCC tumor cells do not differ from the cells of non‐dcSCC. Importantly, desmoplastic melanoma has a better prognosis than other melanoma subtypes, while dcSCC has a worse prognosis than non‐dcSCC.

Several recently published articles and abstracts offer promising glimpses at the biology of dcSCC. Hirakawa et al. found differences in collagen structure and protein expression in both the tumor keratinocytes and the eponymous stroma of dcSCC compared to non‐dcSCC by the application of RNA sequencing and multispectral immunofluorescence [43]. Schmults et al. used spatial transcriptomics to demonstrate that the tumor keratinocytes in dcSCC show gene expression profiles that were dissimilar to non‐dcSCC. The gene expression profiles were intermediate between fibroblasts and keratinocytes [43]. Gomez‐de Castro et al. found statistically significant associations between dcSCC and loss of expression of p21 and phosphorylated S6 ribosomal protein, both of which are involved in cell cycle regulation [44].

Our findings show that dcSCC is a potentially fatal cancer with a significant DSD rate of 12% in the follow‐up periods of these studies (range of medians 19–64 months). To put this in perspective, the overall 5y survival rate for melanoma patients diagnosed in Australia from 2015 to 2019 was 94% [45].

## 6 Conclusion

DcSCC is associated with a higher risk of LR, NM, and DSD compared to non‐dcSCC. The underlying reasons for this aggressiveness are not yet fully understood, though dcSCC is frequently associated with recognized high‐risk features such as immunosuppression, SITM, PNI, larger tumor size, and deeper invasion. DcSCC serves as a key criterion for guiding patients toward more aggressive treatment. Despite all this, there are a limited number of published series and a pressing need for large, prospective, multicentre studies to better understand this potentially life‐threatening malignancy.

## Author Contributions

A.D. conceptualization, formal analysis, investigation, methodology, project administration, writing original draft, writing review and editing; H.M. formal analysis, investigation, writing review and editing; R.D. formal analysis, supervision, writing review and editing; N.B., R.L., D.L. writing review and editing; T.H. writing review and editing.

## Funding

This work was supported by Australia Government, Australian Government Research Training Program Scholarship.

## Disclosure

No AI was used in this paper.

## Ethics Statement

The authors have nothing to report.

## Consent

The authors have nothing to report.

## Conflicts of Interest

The authors declare no conflicts of interest.

## Supporting information


**Data S1:** cup70079‐sup‐0001‐Supinfo.docx.

## Data Availability

The data that support the findings of this study are available from the corresponding author upon reasonable request.
