# Supplementary material for: Desmoplastic Cutaneous Squamous Cell Carcinoma Is an Aggressive and Potentially Fatal Skin Cancer: A Systematic Review
Source: J Cutan Pathol. 2026 Mar 15;53(6):551–61. doi: 10.1111/cup.70079 (PMC13136062; doi:10.1111/cup.70079)
Supplement: Supplementary file 1 — Data S1: cup70079‐sup‐0001‐Supinfo.docx. [file CUP-53-551-s001.docx]

## Supplemental Material


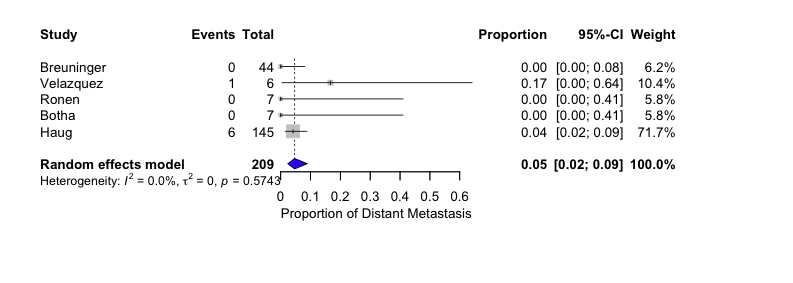


**Supplemental Figure 1. Forrest plot for distant metastasis in the included studies**


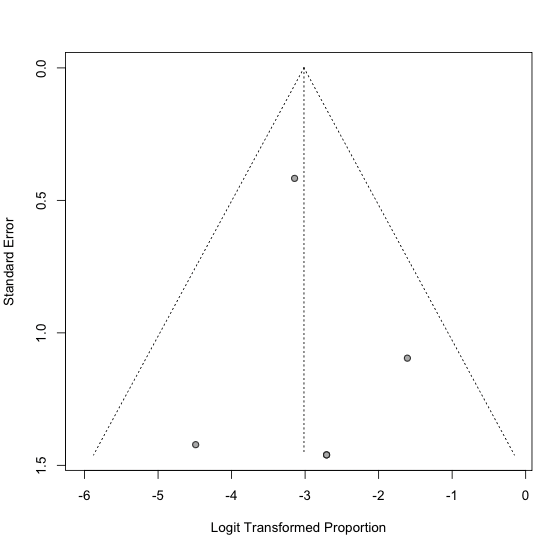


**Supplemental Figure 2. Funnel plot for studies included in pooled proportions in distant metastasis**


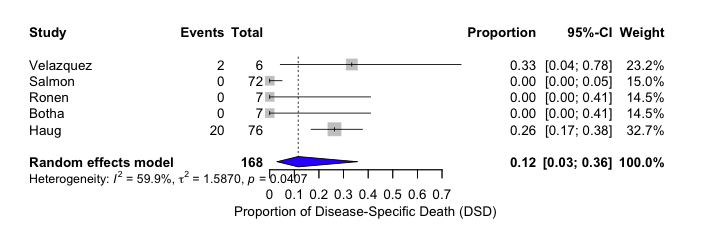


**Supplemental Figure 3. Forrest plot for disease specific death in the included studies**


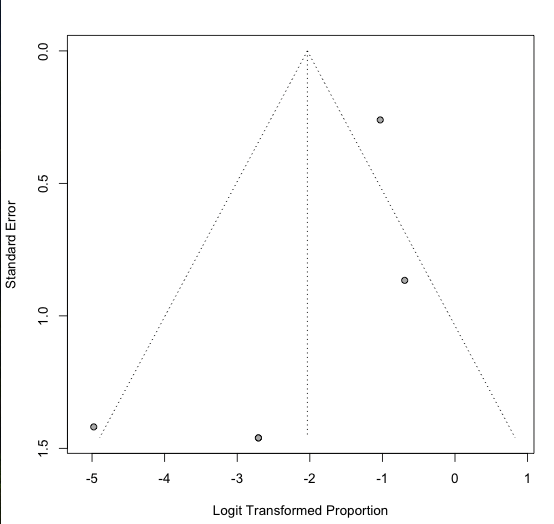


**Supplemental** **Figure 4. Funnel plot for studies included in pooled proportions in disease related deaths**

| CASP Appraisal Question | Breuninger et al., 1996 | Velazquez et al., 2020 | Salmon et al., 2011 | Botha et al., 2018 | Ronen et al., 2028 | Haug et al., 2020 |
| --- | --- | --- | --- | --- | --- | --- |
| Was there a clear aim of the research? | No | No | Yes | Can’t tell | Yes | Yes |
| Was the methodology appropriate? | Yes | Yes | Yes | Yes | Yes | Yes |
| Was the research design appropriate to address the aim(s)? | Can’t tell | Yes | Yes | Yes | Yes | Yes |
| Was the recruitment strategy appropriate? | Can’t tell | Can’t tell | Yes | Yes | Can’t tell | Yes |
| Was the data collected to address the research issue? | Yes | Yes | Yes | Yes | Yes | Yes |
| Has the relationship between researcher and participants been adequately considered? | Can’t tell | Can’t tell | Can’t tell | Can’t tell | Can’t tell | Can’t tell |
| Have ethical issues been taken into consideration? | No | Yes | No | No | Yes | No |
| Was the data analysis sufficiently rigorous? | Yes | Yes | No | Yes | Yes | Yes |
| Is there a clear statement of findings? | Yes | Yes | Can’t tell | Yes | Yes | Yes |
| Will the results help locally? | Yes | Can’t tell | Can’t tell | Yes | Yes | Yes |
| Appraisal summary | Negative: No aims, this was an audit.  Positive: Data analysis is complete and easily presented. | Negative: Unclear why only 6 cases analysed, 50% had immunosuppressive therapy and no follow-up.  Positive: Data analysis is well presented. | Negative: Missing/inadequate data to follow-up. Difficult to interpret which patients had characteristics as results were pooled.  Positive: Comprised 20 years of cases. | Negative: Case series of n=7  Positive: Discusses the value of research and implications of poor prognosis and aggressive invasion. | Negative: Only 4 year period reviewed, not clear why this period was chosen.  Positive: Clear aim, study design, and presentation of results. | Negative: Presentation of manuscript was difficult to follow as results and discussion were presented before methods.  Positive: Large cohort size. |

**Supplemental Table 1. CASP appraisal tool to enable objective insight of studies through a traffic light system** (Yes = green; Can’t tell = orange; No = red)
